# Screening and bioinformatics analysis of a ceRNA network based on the circular RNAs, miRNAs, and mRNAs in pan‐cancer

**DOI:** 10.1002/cam4.3375

**Published:** 2020-08-15

**Authors:** Zhanghan Chen, Jie Huang, Yanling Feng, Zehuan Li, Ying Jiang

**Affiliations:** ^1^ Department of General Surgery Zhongshan Hospital Fudan University Shanghai China; ^2^ Department of Pathology Shanghai Public Health Clinical Center Fudan University Shanghai China; ^3^ Department of General Surgery Xiamen Branch Zhongshan Hospital Fudan University Fujian China

**Keywords:** bioinformatics analysis, ceRNA network, circRNA, pan‐cancer

## Abstract

**Background:**

The pan‐cancer analysis has recently brought us into a novel level of cancer research. Nowadays, the Circular RNAs (circRNAs) is becoming increasingly important in the occurrence and progression of tumors. Nevertheless, the specific expression patterns and functions of circRNAs in the pan‐cancer remains unclear. Here we aimed to explore the expression patterns and functions of circRNAs in pan‐cancer.

**Methods:**

We combined our microarray with seven circRNA arrays from the Gene Expression Omnibus (GEO) database and transcriptome profiles were acquired from The Cancer Genome Atlas (TCGA) database. A circRNA‐miRNA‐mRNA network was created and analyzed using multiple bioinformatic approaches including Gene Ontology (GO), Kyoto Encyclopedia of Genes and Genomes (KEGG) enrichment analysis, Search Tool for the Retrieval of Interacting Genes (STRING) database, cytoHubba and MCODE app. Cell function assays including CCK‐8 analysis, colony formation, and transwell assay were used to explore pan‐circRNAs’ functions.

**Results:**

A panel of 6 circRNAs, 11 miRNAs, and 318 mRNAs was found to be differentially expressed (DE) in pan‐cancer. A circRNA‐miRNA‐mRNA network was also constructed. Then, a circRNA‐miRNA‐hub gene network was created according to 5 pan‐circRNAs, 8 pan‐miRNAs, and 16 pan‐mRNAs. Enrichment analysis pointed out the possible association of DEmRNAs with pan‐cancer is transcriptional misregulation in cancer. Overexpression of hsa_circ_0004639 and silence of hsa_circ_0008310 can inhibit the malignant biological properties of cancer cells.

**Conclusions:**

Six pan‐circRNAs were discovered and their regulating mechanisms were predicted. Those findings together will give a new insight into pan‐cancer research and present potential therapy targeting as well as promising biomarkers.

## Graphical abstract

Bioinformatics analysis of circRNA in pan‐cancer.

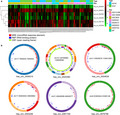

## 1 INTRODUCTION

In recent years, the pan‐cancer analysis brought us into a novel level of cancer research.^1^, ^2^ The commonalities that exist in different types of cancers can help us explore the mechanisms and predict treatment outcomes from one tumor type to another tumor type.^3^ For instance, the synthesis and accumulation of melatonin in the tumor microenvironment were found to be negatively correlated with tumor and mutation burden as well as immunogenic features in 14 solid cancer types, which suggested that melatonin can be a forecaster as well as a predictor of response to immunotherapy.^4^ Tamborero et al analyzed infiltrating immune cell populations in pan‐cancer and discovered that cytotoxic immunophenotypes are negatively correlated with tumors’ malignancy.^5^

It is worth noting that circRNAs is taking an increasingly pivotal part in cancer oncogenesis,^6^ and its special circular structure made it stable enough to serve as an ideal biomarker and therapeutic target in cancers.^7^, ^8^, ^9^ Whereas, the expression profiles and functions of circRNAs in pan‐cancer (pan‐circRNA) remains unclear. Moreover circRNAs can serve as competing endogenous RNAs (ceRNAs) to sponge their targeted miRNAs and thus influence the function of miRNAs and the downstream mRNAs.^10^ circTP63 can sponge miR‐873‐3p and upregulates CENPA and CENPB which facilitate cell cycle progression.^11^ Another research found hsa_circ_0091570 can sponge miR‐1307 to regulate ISM1 expression which works in hepatocellular cancer (HCC) progression.^12^ Here we explored the circRNAs expression and functions in the pan‐cancer.

The study we presented here combined our microarray with multiple GEO arrays. Six DEcircRNAs were identified as pan‐circRNAs to be probably involved in carcinogenesis in pan‐cancer. Then the data of miRNA and mRNA expression profiles were obtained from TCGA to perform the differential analysis in pan‐cancer. GO and KEGG enrichment analysis and PPI networks were used to further explore the function of the pan‐mRNAs. From the pan‐circRNAs, DEmiRNAs in pan‐cancer (pan‐miRNAs), and DEmRNAs in pan‐cancer (pan‐mRNAs), the circRNA‐miRNA‐mRNA network was constructed to help us find their potential functions in pan‐cancer.

## 2 MATERIALS AND METHODS

### 2.1 Selection and profiling of RNA datasets

All the eligible circRNAs microarray datasets were downloaded from GEO. The selection criteria were as follows: (a) Inclusion of datasets included cancer tissue and adjacent normal tissues; (b) Arrays contained a minimum of six tumors and six adjacent normal tissue samples; (c) Array analyses of human solid cancer samples. Finally, seven datasets (GSE79634, GSE83521, GSE90737, GSE93522, GSE97332, GSE101586, and GSE126095) were included (Table 1). The basic information of the enrolled patients was listed in Table S1. Besides, eight types of cancers’ transcriptome profiles (PAAP, BRAC, STAD, CESC, THCA, LIHC, LUAD, and COAD) were acquired from TCGA. R software was used to calibrate, standardize, and log_2_ transform the downloaded files.

**TABLE 1:** Basic information of seven eligible arrays

ID	Platform	Type	Uploader	Year	Samples (N/T)
GSE79634	GPL19978	Pancreatic ductal adenocarcinoma	Wang	2016	20/20
GSE83521	GPL19978	Gastric cancer	Zhang	2017	6/6
GSE90737	GPL22722	Cervical tumor	Li	2018	10/10
GSE93522	GPL19978	Papillary thyroid carcinoma	Peng	2017	6/6
GSE97332	GPL19978	Hepatocellular carcinoma	Han	2017	7/7
GSE101586	GPL19978	Lung adenocarcinoma	Qiu	2017	5/5
GSE126095	GPL19978	Colorectal cancer	Chen	2019	10/10

### 2.2 Samples collection and ceRNA microarray

All six patients were selected from Zhongshan Hospital of Fudan University (Shanghai, China) and signed the informed consent. Our study was approved by the Ethics Committee of Zhongshan Hospital and the information of these six patients has been reported in our previous work.^13^ The cancer tissues and paired normal tissues’ RNAs were extracted using RNAsimple Total RNA Kit (TIANGEN, Germany), then quantified by DS‐11 spectrophotometer (DeNovix, USA). Agilent SBC circRNA microarray was applied to explore the expression profiles of our samples. The process can be simply concluded in RNA amplification, labeling, purification, hybridized with microarray and scanned in images.

### 2.3 Identification of the differentially expressed circRNAs, miRNAs and mRNAs

Batch effects were batch normalized using sva package. Limma package was introduced to perform differential analysis. The threshold was set as |log_2_FC | over 1 and adjusted P‐value less than 0.05 for finding DEcircRNAs. Besides, the edgeR package was applied to find DEmiRNAs and DEmRNAs with thresholds of adjusted P‐value less than 0.05, |log_2_FC | over 1 for DEmiRNAs and over 2 for DEmRNAs.

### 2.4 GO enrichment analysis and KEGG analysis

GO was used to identify the enriched terms based on the pan‐mRNAs in the ceRNA network to find the possible roles of these pan‐circRNAs. KEGG was applied to find the enriched pathways. ClusterProfiler package was used to perform these analyses. Significant terms and pathways (P‐value over 0.05) were visualized using the ggplot2 package.

### 2.5 Formation of the circRNA–miRNA–mRNA network

Databases including Circular RNA Interactome database, MicroRNA Target Prediction Database, RegRNA 2.0 database, and the Cancer‐Specific CircRNA Database were used to explore potential downstream miRNAs of DEcircRNAs. The predicted miRNAs were compared to DEmiRNAs based on TCGA. And miRNAs that overlapping at least two databases (included TCGA database) were selected as candidates. Next, databases including miRTarBase database, microRNA Target Prediction Database, TargetScan database, and starbase database were used to find possible targeted mRNA of miRNA. The predicted mRNAs were intersected with DEmRNAs based on TCGA. And mRNAs that overlapping at least three databases (TCGA database included) were selected as candidates.

### 2.6 Formation of protein‐protein interaction (PPI) network and identification of key modules

**FIGURE 1:**
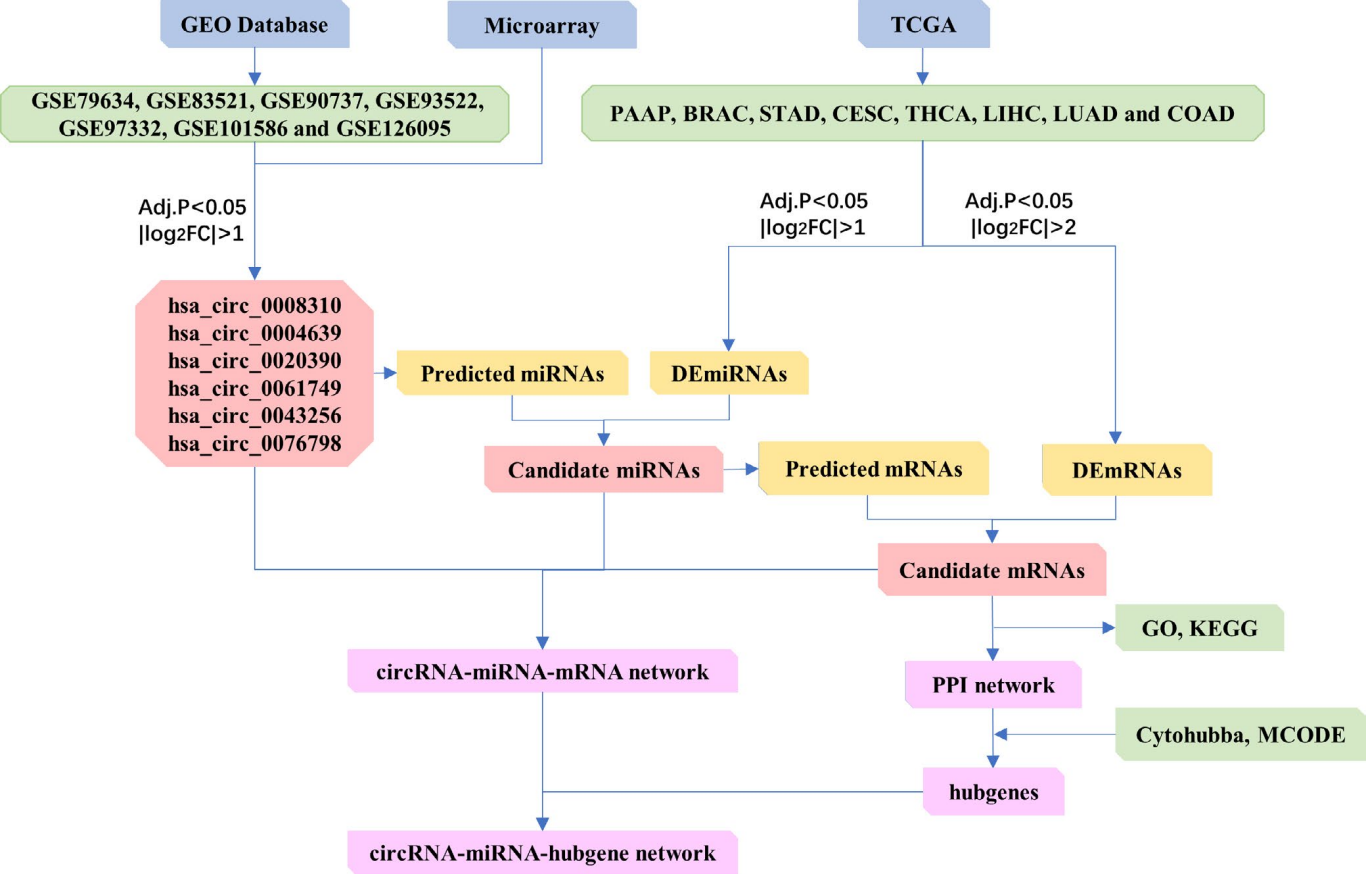
Flowchart of the construction of ceRNA networks

The PPI network consisted of pan‐mRNAs were constructed by the STRING online tool. The filter was set as the interaction score of >0.9 and excluded disconnected nodes for a PPI network. We then combined the cytoHubba and MCODE apps in Cytoscape v3.6.1 to select hub genes with the criteria degree >4, MCODE score >10. The workflow is presented in Figure 1.

### 2.7 Cell culture

The breast cancer cells T47D, MCF‐7, ZR‐75‐1, MDA‐MB‐453, MDA‐MB‐231, MDA‐MB‐468, Hs‐578T, liver cancer cells PLC/PRF/5, Hep G2, HCCLM3, MHCC97H, colon cancer cell HRT18, pancreatic cancer cell PANC‐1, lung cancer cell A549, prostate cancer cells PC3, DU145, and normal control cells HBL‐100, HPDE, WRL68 were cultured by DMEM, 1640, F12, or MEM medium with 10% FBS following the instructions.

### 2.8 QRT‐PCR and data analysis

The ProFlex^TM^ PCR system (ABI, USA) and PrimeScript^TM^ RT reagent Kit with gDNA Eraser (TaKaRa, RR047A) were used to get cDNAs. ABI QuantStudio 5 (ABI, USA) and SYBR Premix Ex Taq II (TaKaRa, RR820A) were applied to quantify circRNAs and miRNAs’ expression. The primers are listed in Table S2. 18S and U6 were set as the internal reference for circRNAs and miRNAs, respectively. The 2^−ΔΔCt^ method was used to analyze the results.

### 2.9 Construction and transfection of plasmid and siRNA

To overexpress hsa_circ_0004639, the plasmid contains the whole sequence of hsa_circ_0004639 was produced by Geneseed (Guangzhou, China). To silence hsa_circ_0008310 the siRNA was synthesized by RIBOBIO (Guangzhou, China). X‐tremeGENE HP DNA Transfection Reagent (Roche, Switzerland) was used to transfect plasmid and siRNA.

### 2.10 Cell proliferation and colony formation assay

CCK‐8 (BBI, China) method was applied to estimate cell proliferative potential. We seeded 2000 cells per well in 96‐well plates. Ten microliter of CCK8 liquid was added at 0, 24, 48, and 96 hours, incubated the plate without light at 37°C about 1 hour, then tested OD values by Epoch 2 microplate reader. (BioTek, USA). In terms of cells’ colony formation ability, 2000 cells were added in each well. After 14 days of cell culture, the culture medium was discarded, 500 μL of 4% paraformaldehyde and crystal violet for each well were used to fix and stain the cells, and the cell counting was performed by Image J software.

### 2.11 Migration and invasion assays

To test cell migration ability, each upper chamber (Biofil, China) was seeded 50 000 cells with serum‐free culture medium. Also, to evaluate cell invasion ability, the upper chamber with 1:7 concentration of Matrigel membrane (BD, USA) was used. The lower chambers were infiltrated in a complete culture medium. Cultured about 72 hours the chambers were collected, using the same way described above to fix and stain the cells, then wiped cells fixed in the upper chamber with a swab.

## 3 RESULTS

### 3.1 Identification of differentially expressed genes in pan‐cancer

**FIGURE 2:**
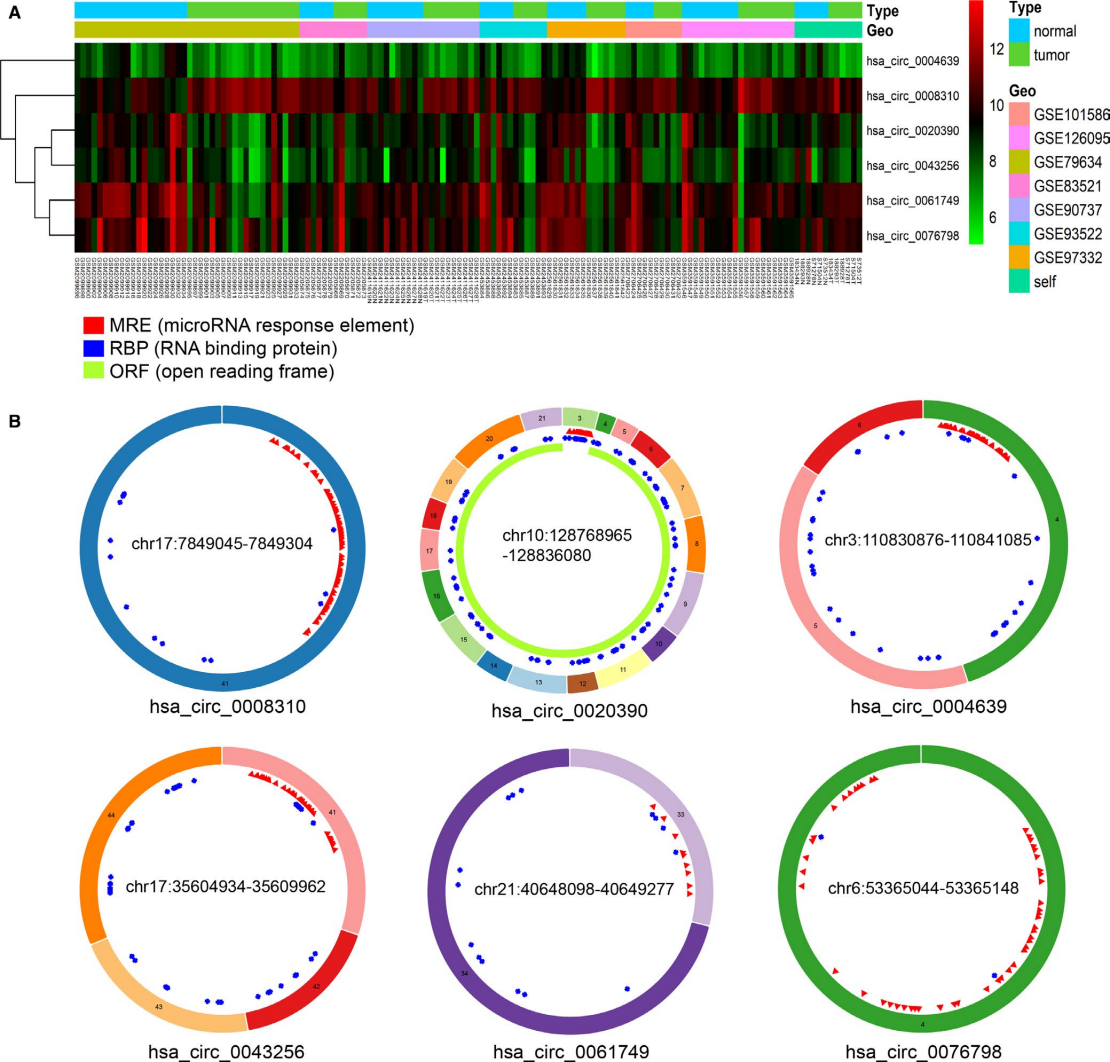
Six pan‐circRNAs and their structures. A, Heatmap of six pan‐circRNAs of the microarrays. B, The structures of six pan‐circRNAs in the CSCD database

**FIGURE 3:**
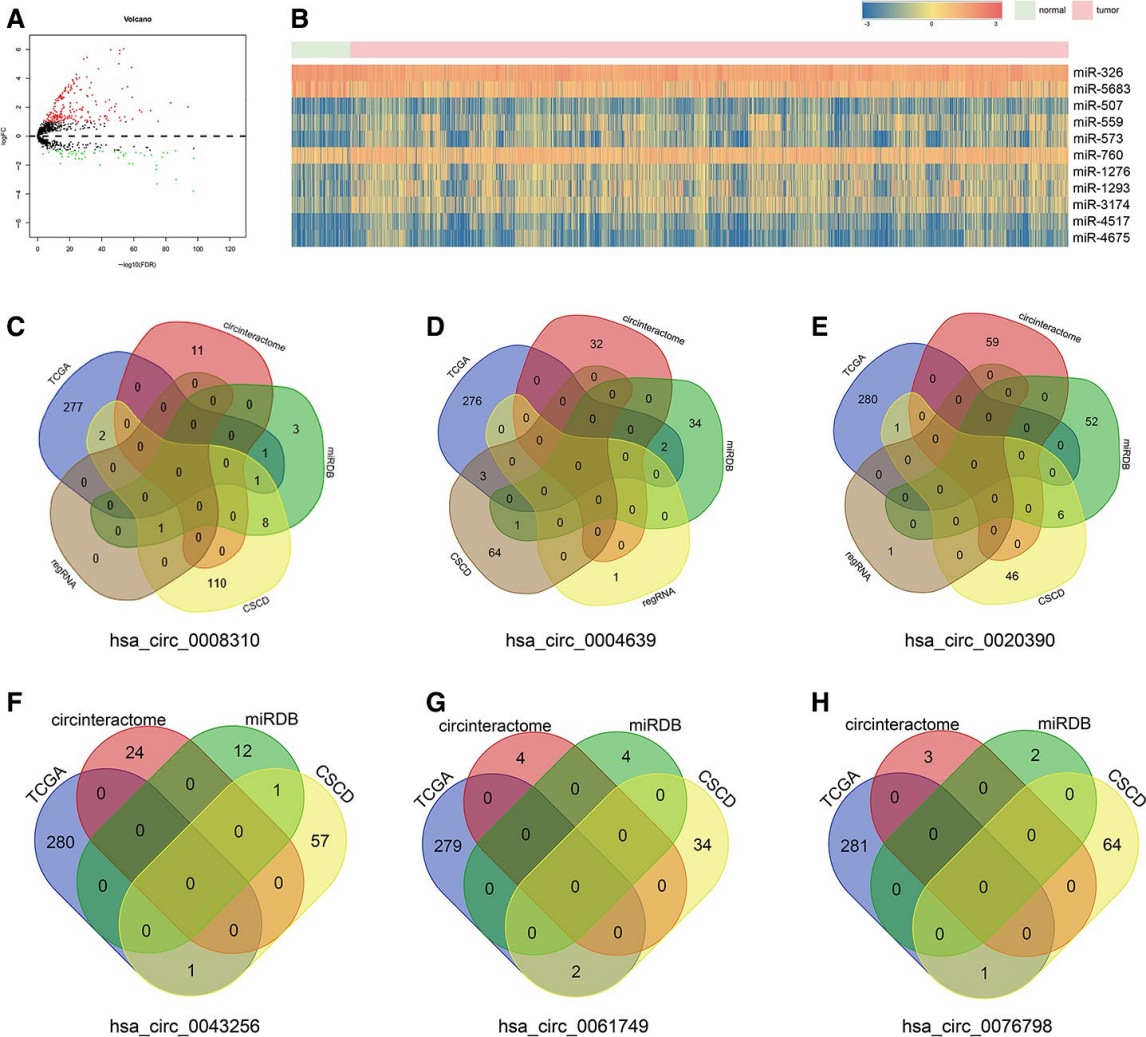
Eleven predicted pan‐miRNAs and their expression. A, The volcano plot for pan‐miRNAs from TCGA. B, The heatmap for candidate pan‐miRNAs expression from TCGA. C‐H, The Venn plots of predicted miRNAs based on six pan‐circRNAs

Six pan‐circRNAs were screened out from multiple microarray datasets, including one upregulated pan‐circRNA (hsa_circ_0008310) and five downregulated pan‐circRNAs (hsa_circ_0020390, hsa_circ_0004639, hsa_circ_0043256, hsa_circ_0061749, and hsa_circ_0076798) (Figure 2A). The information and structures of these six circRNAs from circbase and CSCD databases are shown in Table 2 and Figure 2B. The miRNAs and mRNAs expression profiles of eight cancers were obtained from the TCGA database. The miRNAs data includes 3897 patients and 319 normal control, the mRNAs data include 3860 patients and 360 normal control. We identified 281 pan‐miRNAs (214 upregulated and 67 downregulated) (Figure 3A) and 1610 pan‐mRNAs (1027 upregulated and 583 downregulated). Among them, the most upregulated miRNA is miR‐105‐1 and the most downregulated miRNA is miR‐133b, the most upregulated mRNA OR4D8P he most downregulated mRNA MYH2. Part of up‐ and downregulated pan‐miRNAs and pan‐mRNAs are listed in Table 3.

**TABLE 2:** Basic information of six pan‐circRNAs

ID	Position	Spliced length	Strand	Host gene	Basic level
hsa_circ_0008310	chr17:7849045‐7849304	259	+	CNTROB	Up
hsa_circ_0004639	chr3:110830876‐110841085	757	+	NECTIN3	Down
hsa_circ_0020390	chr10:128768965‐128836080	1901	+	DOCK1	Down
hsa_circ_0061749	chr21:40648098‐40649277	142	−	BRWD1	Down
hsa_circ_0076798	chr6:53365044‐53365148	104	−	GCLC	Down
hsa_circ_0043256	chr17:35604934‐35609962	483	−	ACACA	Down

**TABLE 3:** Top 10 down‐ and upregulated pan‐miRNAs and pan‐mRNAs

Category	Log_2_ (foldchange)	*P*‐value	FDR
Pan‐miRNAs			
miR‐133b	−3.8557	<.0001	<0.0001
miR‐206	−3.8029	<.0001	<0.0001
miR‐133a‐1	−3.7146	<.0001	<0.0001
miR‐133a‐2	−3.7012	<.0001	<0.0001
miR‐1‐2	−3.4271	<.0001	<0.0001
miR‐1‐1	−3.4061	<.0001	<0.0001
miR‐490	−3.3029	<.0001	<0.0001
miR‐208b	−3.0017	<.0001	<0.0001
miR‐486‐1	−2.9522	<.0001	<0.0001
miR‐486‐2	−2.9518	<.0001	<0.0001
miR‐373	4.2734	<.0001	<0.0001
miR‐1269a	4.6588	<.0001	<0.0001
miR‐4652	4.7563	<.0001	<0.0001
miR‐552	5.0074	<.0001	<0.0001
miR‐371a	5.3397	<.0001	<0.0001
miR‐1269b	5.4559	<.0001	<0.0001
miR‐767	5.7066	<.0001	<0.0001
miR‐105‐2	5.9300	<.0001	<0.0001
miR‐372	5.9765	<.0001	<0.0001
miR‐105‐1	6.0400	<.0001	<0.0001
Pan‐mRNAs			
MYH2	−7.5566	<.0001	<0.0001
MYL2	−7.2723	<.0001	<0.0001
MYL1	−7.0817	<.0001	<0.0001
MYH7	−7.0228	<.0001	<0.0001
CKM	−6.8323	<.0001	<0.0001
XIRP2	−6.5619	<.0001	<0.0001
ACTA1	−6.5578	<.0001	<0.0001
CSRP3	−6.3666	<.0001	<0.0001
MIR1‐1HG	−6.2213	<.0001	<0.0001
CA1	−6.1935	<.0001	<0.0001
CASP14	7.2968	<.0001	<0.0001
MAGEA4	7.4652	<.0001	<0.0001
MAGEA12	7.5404	<.0001	<0.0001
AMY2A	7.5808	<.0001	<0.0001
MAGEC2	7.6100	<.0001	<0.0001
COX7B2	7.7264	<.0001	<0.0001
SOD1P1	7.7715	<.0001	<0.0001
MAGEA6	7.8126	<.0001	<0.0001
DCAF4L2	7.9242	<.0001	<0.0001
OR4D8P	11.2253	<.0001	<0.0001

### 3.2 Formation of the ceRNA network

**FIGURE 4:**
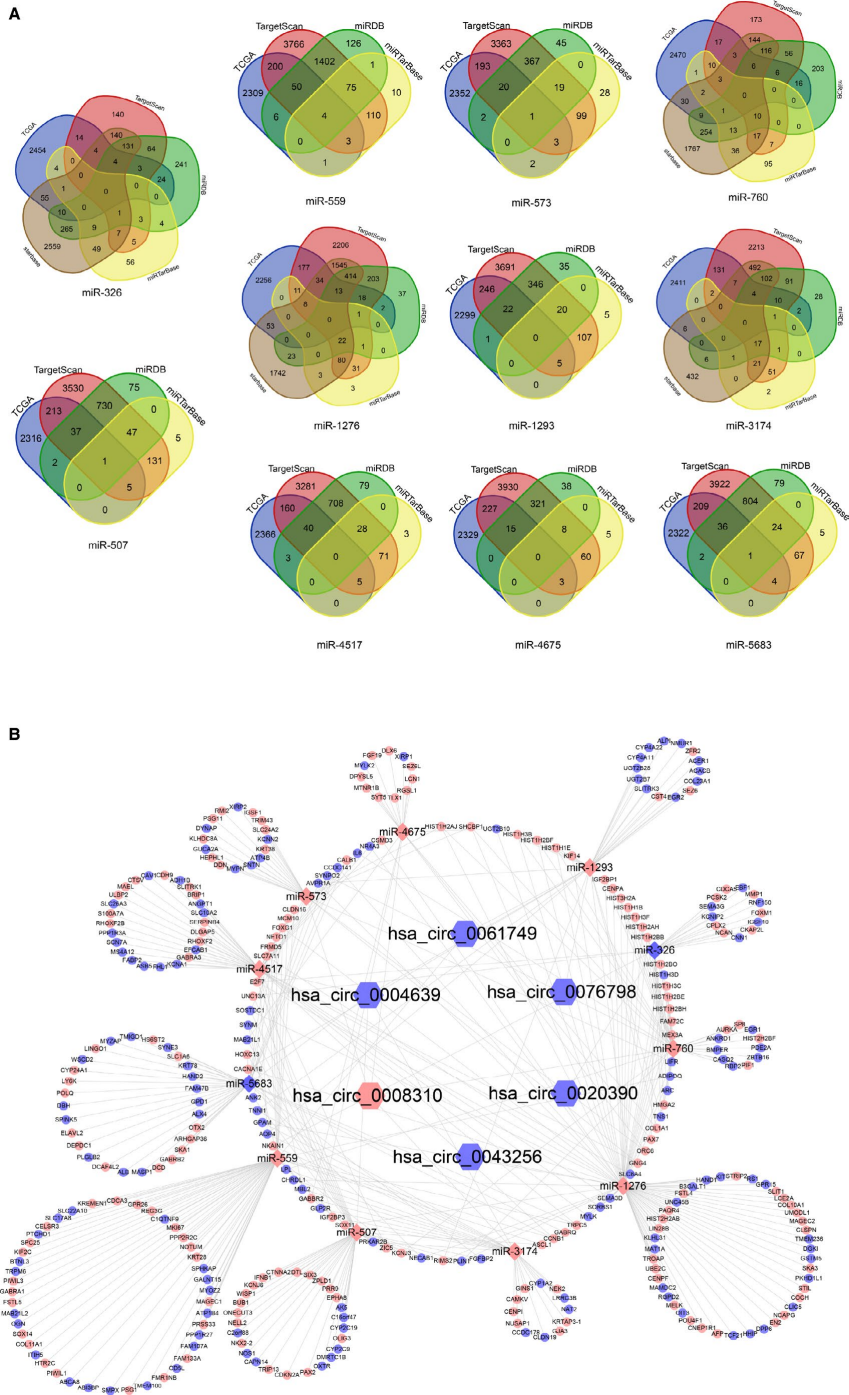
Downstream candidate pan‐mRNAs analysis and preliminary circRNA‐miRNA‐mRNA for the 6 pan‐circRNAs, the 11 pan‐miRNAs, and the 318 pan‐mRNAs. A, The Venn plots of predicted mRNAs based on 11 pan‐miRNAs. B, Preliminary circRNA‐miRNA‐mRNA network the six pan‐circRNAs, the 11 pan‐miRNAs, and the 318 pan‐mRNAs. Color in red refers to up‐regulated, color in blue refers to downregulated

All six pan‐circRNAs were searched in the circinteractome, miRDB, RegRNA 2.0, and CSCD databases to predict their targeted miRNAs. The predicted miRNAs were compared to pan‐miRNAs from TCGA (Figure 3C‐H). After intersecting with the pan‐miRNAs, only 11 pan‐miRNAs remained, which include two downregulated pan‐miRNAs (miR‐326 and miR‐5683) and nine upregulated pan‐miRNAs (miR‐507, miR‐559, miR‐573, miR‐760, miR‐1276, miR‐1293, miR‐3174, miR‐4517, and miR‐4675) (Figure 3B). Next, we used miRTarBase, miRDB, TargetScan, starbase and TCGA databases to predict potential downstream mRNA of miRNA, 318 pan‐mRNAs were selected as candidate genes in total (Figure 4A). Finally, a pan‐cancer associated ceRNA network was constructed (Figure 4B).

### 3.3 Formation of PPI network and key modules identification

**FIGURE 5:**
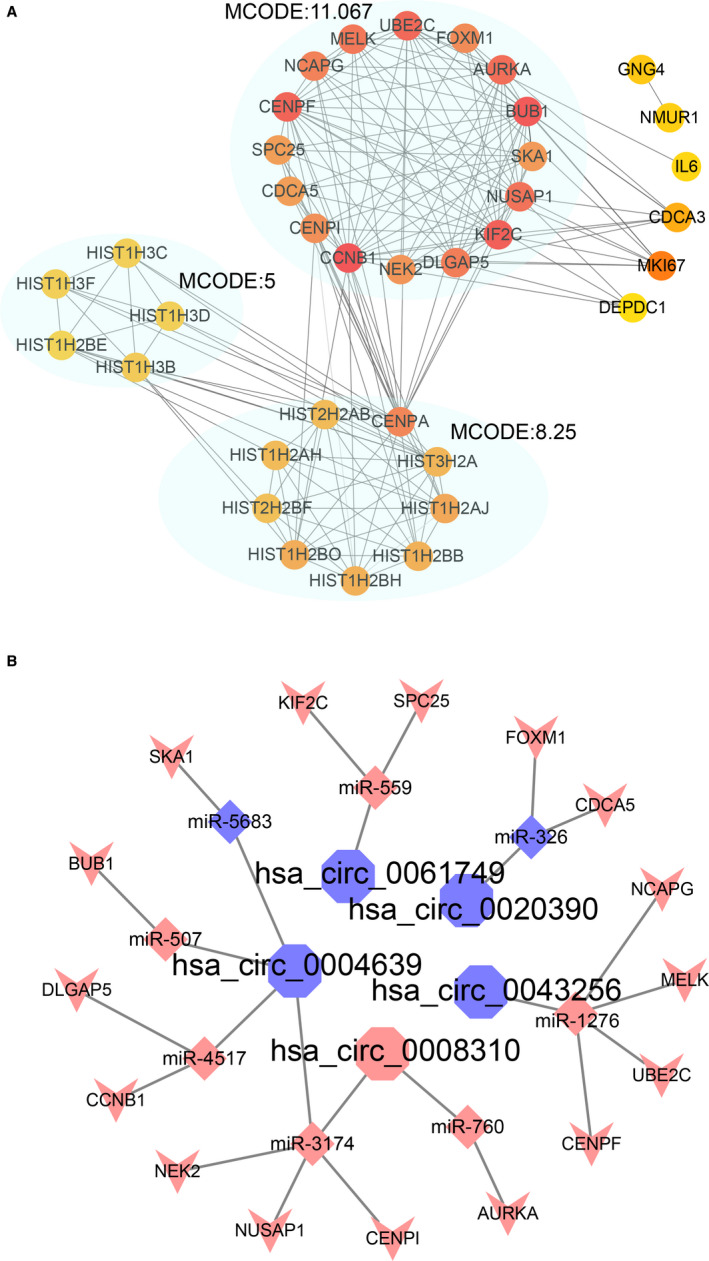
CircRNA‐miRNA‐hub genes network for the 6 pan‐circRNAs, the 11 pan‐miRNAs, and the 318 pan‐mRNAs. A, PPI network for 318 genes. Each ellipse represents the cluster identified by the MCODE algorithm. the gradation of color was identified by Cytohubba. B, The circRNA‐miRNA‐hub genes network for the 5 circRNAs, 8 miRNAs, and 16 mRNAs. Color in red refers to upregulated, color in blue refers to downregulated

We analyzed 318 pan‐mRNAs by STRING with single nodes excluded and the threshold as 0.9. To identify hub genes, the result was downloaded from the STRING and analyzed in Cytoscape. Then, a 36‐nodes‐and‐181‐edges network was identified, which included three modules. And the highest‐MCODE‐scoring module contained 16 hub genes (CDCA5, NCAPG, CENPI, DLGAP5, AURKA, SKA1, UBE2C, KIF2C, BUB1, CENPF, CCNB1, MELK, SPC25, FOXM1, NUSAP1, NEK2) was selected (Figure 5A). Finally, we also constructed a network based on 5 circRNAs (hsa_circ_0008310, hsa_circ_0020390, hsa_circ_0004639, hsa_circ_0043256, and hsa_circ_0061749, except for the hsa_circ_0076798 that has no connection with the hub genes), 8 miRNAs (miR‐326, miR‐5683, miR‐507, miR‐559, miR‐760, miR‐1276, miR‐3174, and miR‐4517), and 16 hub genes (Figure 5B).

### 3.4 GO and pathway enrichment analysis

**FIGURE 6:**
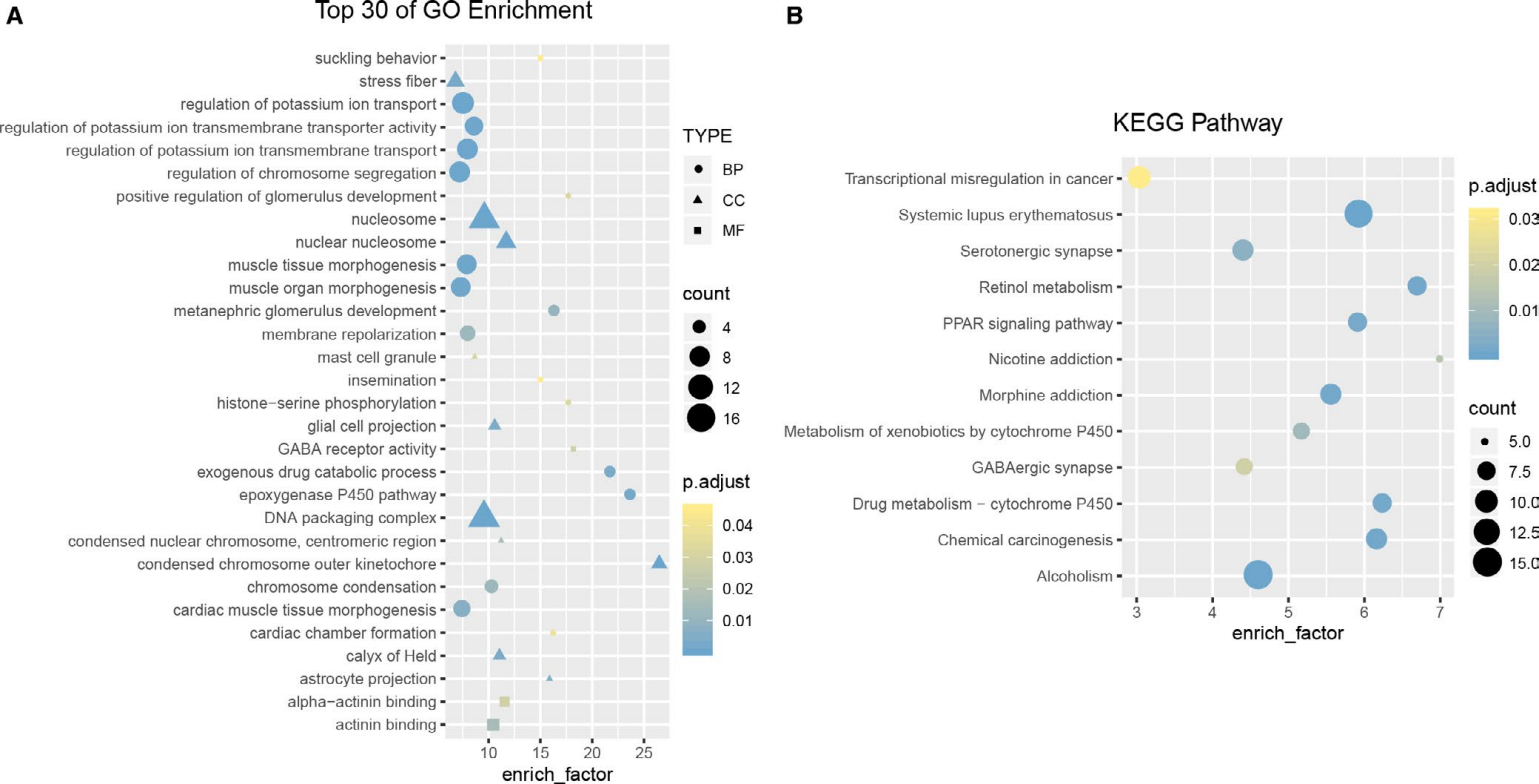
Top 30 GO terms and 12 KEGG pathways enriched by pan‐mRNAs. Bubble plots of the GO (A) and KEGG (B) analysis based on candidate pan‐mRNAs from TCGA

GO and KEGG enrichment analysis was performed over the 318 overlapped genes. For the Biology Process, the pan‐mRNAs were enriched in the epoxygenase P450 pathway with an enriching factor of 23.5. On Cell Component, the condensed chromosome outer kinetochore has the highest enrich factor of 26.5. In terms of Molecular Function, GABA receptor activity with enrich factor of 18.2. Pathway enrichment analysis indicated that most pan‐mRNAs were enriched in drug metabolism‐cytochrome P450, chemical carcinogenesis, PPAR signaling pathway, metabolism of xenobiotics by cytochrome P450 and transcriptional misregulation in cancer (Figure 6A and B).

### 3.5 Expression of hsa_circ_0004639 and hsa_circ_0008310 circular RNA in pan‐cancer cells

**FIGURE 7:**
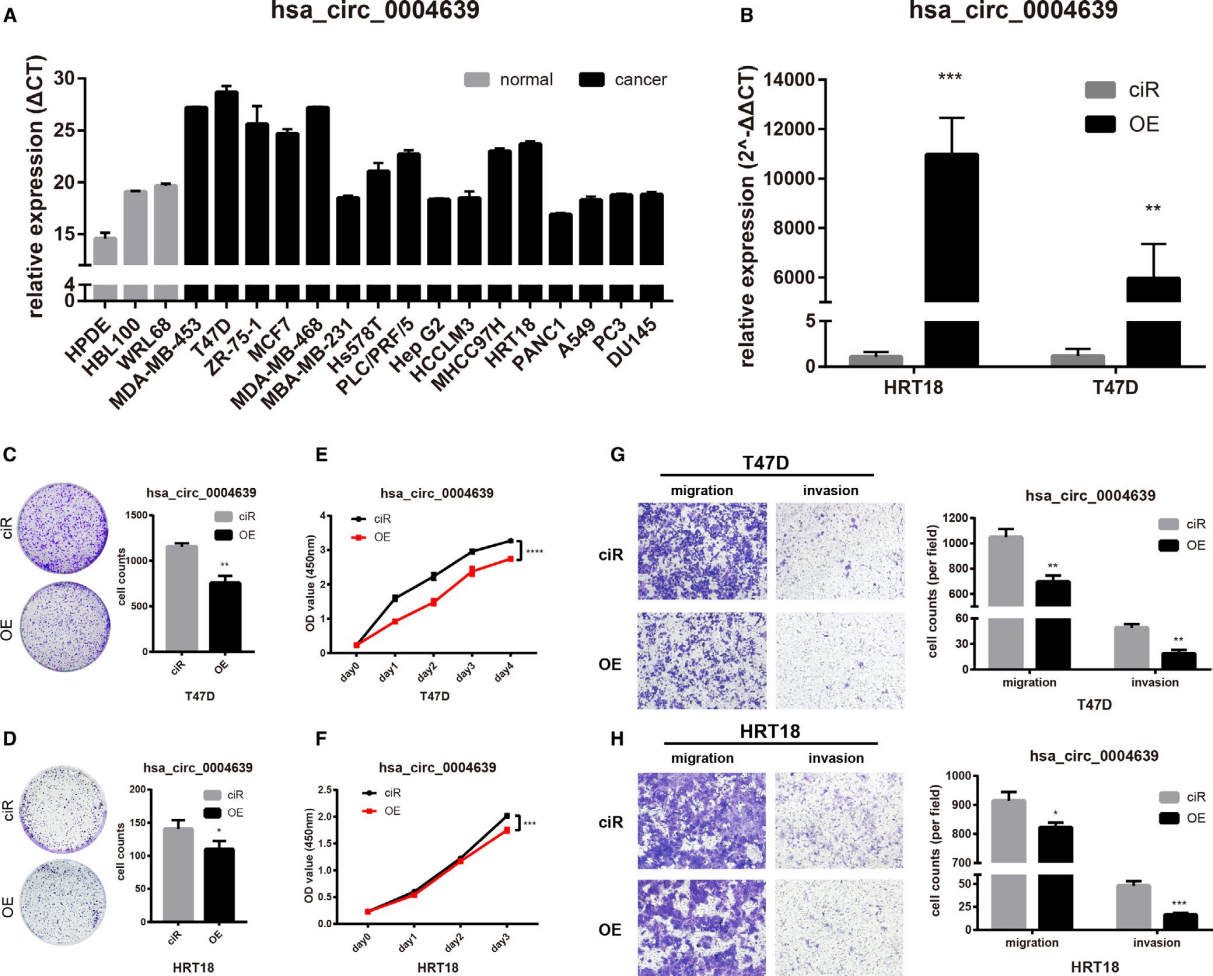
Hsa_circ_0004639 inhibits cancer cells proliferation, migration, and invasion. A, Basic levels of hsa_circ_0004639 in our cell line panel. B, The transfection efficiency of the plasmid in T47D and HRT18. C and D, Colony formation assays of transfected T47D and HRT18. E and F,CCK8 analysis of transfected T47D and HRT18. G and H, Migration and invasion assays of transfected T47D and HRT18. Scale bar = 200 μm. *P < .05, **P < .01, ***P < .001

**FIGURE 8:**
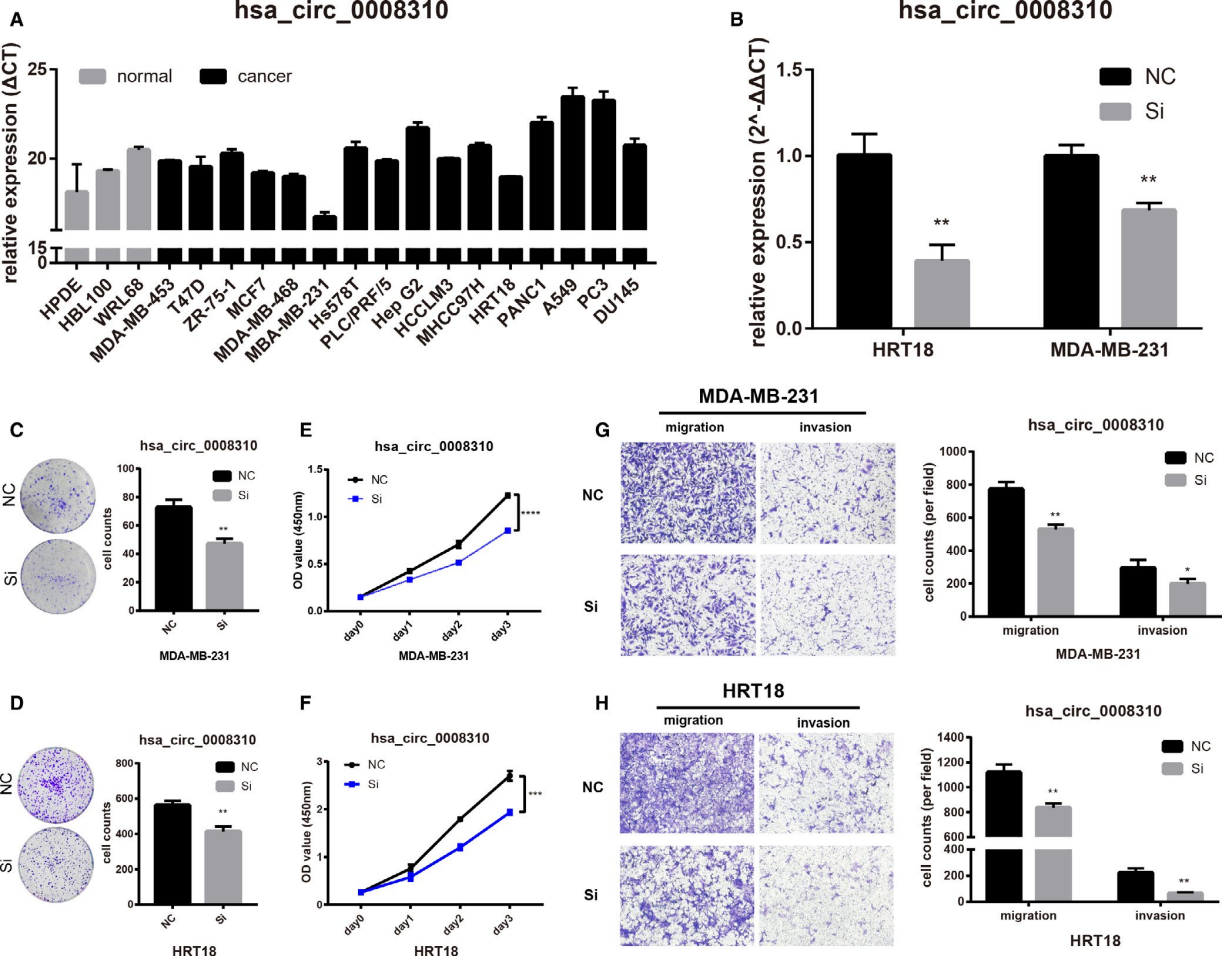
Hsa_circ_0008310 promotes cancer cells proliferation, migration, and invasion. A, Basic level of hsa_circ_0008310 in our cell line panel. B, The transfection efficiency of siRNA in MDA‐MB‐231 and HRT18. C and D, Colony formation assays of MDA‐MB‐231 and HRT18. E and F, CCK8 analysis of MDA‐MB‐231 and HRT18. G and H, Migration and invasion assays for MDA‐MB‐231 and HRT18

We tested hsa_circ_0004639 and hsa_circ_0008310 levels in 19 cell lines, including breast, liver, lung, pancreas, colon, and prostate cancer cell lines as well as three normal cell lines (breast, liver, and pancreas). Hsa_circ_0004639 levels are downregulated in most cancer cell lines which is similar to what we predicted. However, the level of hsa_circ_0008310 in cancer cell lines is slightly different from what we supposed (Figures 7A and 8A).

### 3.6 Overexpression of hsa_circ_0004639 can inhibit cancer cells’ proliferation, migration, and invasion

Overexpression plasmid was successfully transfected and expressed in T47D and HRT18 (Figure 7B). The colony formation assays indicated that the colony‐forming ability of the cells was significantly decreased by overexpressing hsa_circ_0004639 (Figure 7C and D). In cell proliferation assays, the upregulation of hsa_circ_0004639 significantly inhibited the growth of T47D as well as HRT18 (Figure 7E and F). Besides, the migration and invasion abilities of cancer cells were also inhibited by hsa_circ_0004639 overexpression (Figure 7G and H).

### 3.7 Silence of hsa_circ_0008310 can inhibit cancer cells’ proliferation, migration, and invasion

The siRNA of hsa_circ_0008310 was successfully transfected into MDA‐MB‐231 and HRT18 (Figure 8B). Colony formation assays indicated that the colony‐forming ability of the cells was significantly decreased by silencing hsa_circ_0008310 (Figure 8C and D). In cell proliferation assays, the silence of hsa_circ_0008310 also inhibited the growth of MDA‐MB‐231 as well as HRT18 (Figure 8E and F). Moreover the migration and invasion abilities of cancer cells were also significantly inhibited by hsa_circ_0008310 down‐expression (Figure 8G and H).

### 3.8 Validation of downstream miRNAs of hsa_circ_0004639 and hsa_circ_0008310

**FIGURE 9:**
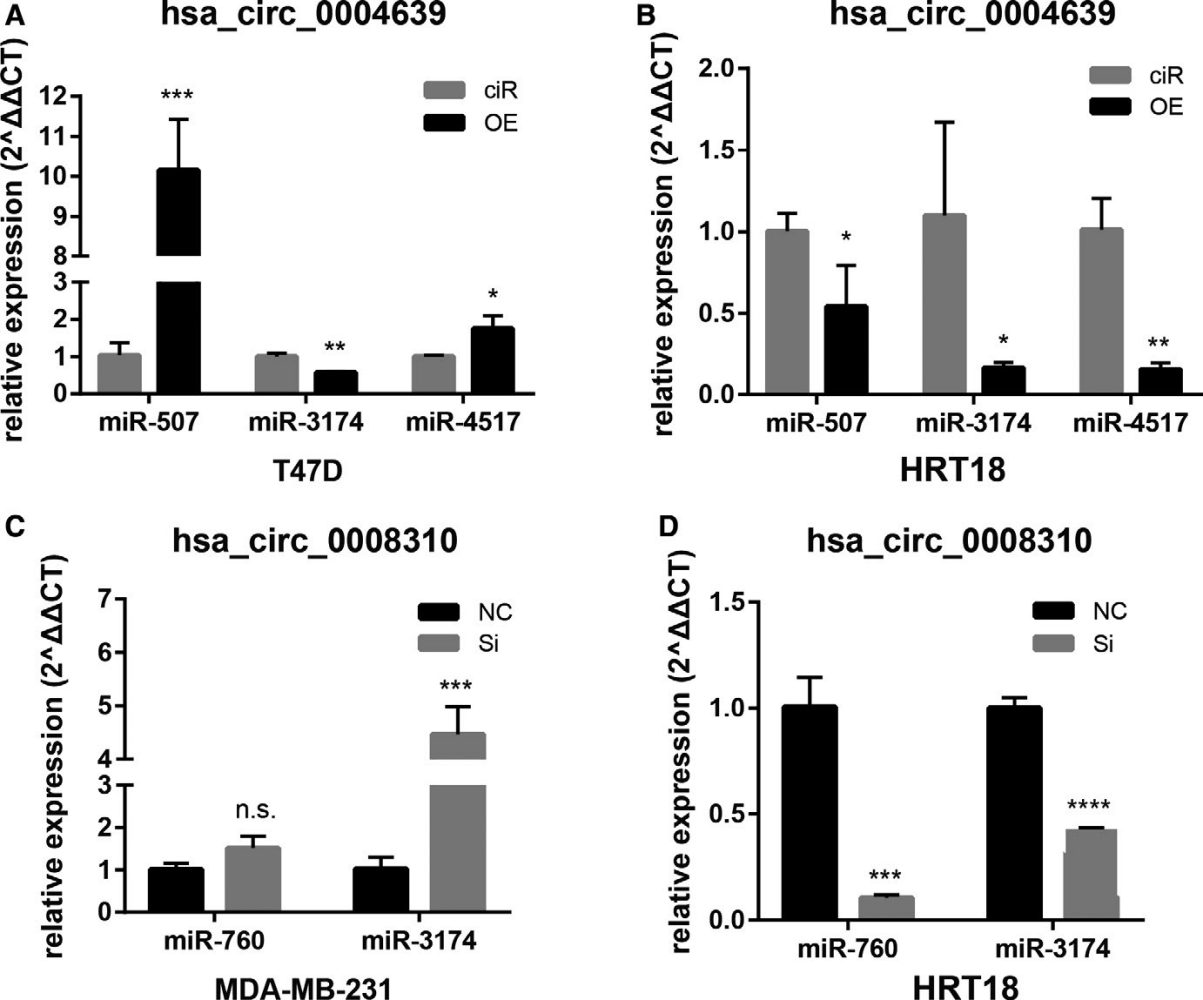
Identification of downstream miRNAs. A and B, the expression of miR‐507, miR‐4517, and miR‐3174 in T47D and HRT18 transfected with hsa_circ_0004639 expression vector and mock. C and D, the expression of miR‐760 and miR‐3174 in MDA‐MB‐231 and HRT18 transfected with hsa_circ_0008310 siRNA and mock

Furthermore, downstream pan‐miRNAs in Figure 5 were tested. The results showed that overexpression of hsa_circ_0004639 in T47D had increased the level of miR‐507 and miR‐4517 and decreased the level of miR‐3174 (Figure 9A). Meanwhile, the overexpression of hsa_circ_0004639 in HRT18 had significantly decreased the expression of all miR‐507, miR‐3174, and miR‐4517 (Figure 9B). By silencing the hsa_circ_0008310, MDA‐MB‐231 had a significantly increased level of miR‐3174 (Figure 9C) while HRT18 had a significantly decreased level of both miR‐760 and miR‐3174 (Figure 9D). Only miR‐3174 was consistently decreased in both T47D and HRT18 when hsa_circ_0004639 was overexpressed.

## 4 DISCUSSION

During the past centuries, researches on a single cancer were booming. However, in recent years, scientists found that between different types of cancers there exist some similarities.^14^ And by putting multiple cancers together we can better understand the features of cancers.^2^, ^15^ CircRNAs play a vital role in cancer occurrence and development, and their stable circular structure laid the foundation for it to become a potential biomarker and therapeutic target.^7^, ^8^, ^9^ In this study, we searched circRNA microarrays in GEO datasets for the seven kinds of tumors and integrated them with our BC microarray data. After combination, normalization and differential analysis of these eight arrays, only six circRNAs were screened out with significant difference. Considering the heterogeneity between different types of tumors, to better characterize the features of cancer, these six circRNAs were all included for further analysis. Survival analysis based on TCGA databases showed that most of the host genes of them were related to patients' prognosis (Figure S1). Among these pan‐circRNAs, hsa_circ_0043256 was previously founded to mediate cinnamaldehyde induced cell apoptosis in non‐small cell lung cancer.^16^

The endogenous competition mechanism maintains the circRNAs can sponge miRNAs to influence their functions.^10^ Therefore, we explore the potential ceRNA networks of these six pan‐circRNAs. Since circRNAs can also act as a protector or promoter of miRNAs, not all circRNAs and miRNAs were inversely correlated.^17^, ^18^ Among the 281 pan‐miRNAs, two downregulated miRNAs miR‐326 and miR‐5683 and nine upregulated miRNAs miR‐507, miR‐559, miR‐573, miR‐760, miR‐1276, miR‐1293, miR‐3174, miR‐4517, and miR‐4675 were screened out as targeted pan‐miRNAs. These 11 miRNAs were predicted to target 318 mRNAs. Further, a PPI network was formed based on 318 mRNAs. With the help of Cytoscape's apps, 16 hub genes (CDCA5, NCAPG, CENPI, DLGAP5, AURKA, SKA1, UBE2C, KIF2C, BUB1, CENPF, CCNB1, MELK, SPC25, FOXM1, NUSAP1, and NEK2) were identified. Then, a new ceRNA network was formed, which includes 5 circRNAs (hsa_circ_0008310, hsa_circ_0020390, hsa_circ_0004639, hsa_circ_0043256, and hsa_circ_0061749, except for the hsa_circ_0076798 that has no connection with the hub genes), 8 miRNAs (miR‐326, miR‐5683, miR‐507, miR‐559, miR‐760, miR‐1276, miR‐1293, miR‐3174, and miR‐4517) and 16 hub genes. We tried to analyze the common features of these pan‐circRNAs. Interestingly, both hsa_circ_0004639 and hsa_circ_0008310 are supposed to regulated miR‐3174 and thus may further regulated NUSAP1, NEK2, and CENPI. We also analysis the sequence of all six circRNAs and found that all of these pan‐circRNAs are exonic circRNAs. What's more, all these pan‐circRNAs obtain ORF (open reading frame). Notably, the hsa_circ_0004639 and hsa_circ_0008310 also obtain IRES (Internal Ribosome Entry Site), which indicates their potential of translating peptides or proteins (Table S3).

The GO and KEGG enrichment analysis found the 318 mRNAs were involved in multiple biological functions associated with cancers. Specifically, these mRNAs were enriched in chromosome segregation, chemical carcinogenesis, histone‐serine phosphorylation, DNA packaging complex and transcriptional misregulation in cancer and thus relating to cancer occurrence and development.^19^, ^20^ Besides, the enrichment in exogenous drug catabolic process, metabolism of xenobiotics by cytochrome P450, drug metabolism‐cytochrome P450 as well as drug resistance and metabolism indicate that those mRNAs were closely related to drug resistance.^21^, ^22^ The network we constructed is closely related to the occurrence and development as well as drug resistance of cancers. In accordance with our analysis, a few reports showed that the predicted pan‐miRNAs and targeted mRNAs in this study contribute to the tumorigenesis and drug resistance of cancers.^23^, ^24^, ^25^, ^26^, ^27^ For example, miR‐326 function as a tumor suppressor,^28^ which involved reversing chemoresistance ^29^ and inhibition of proliferation,^30^ invasion ^31^ and metastasis ^32^ in cancers. Its’ predicted target FOXM1 is a transcriptional activator involved in cell proliferation, which is closely connected with tumorigenesis.^33^ And NEK2 which was predicted targeted by miR‐3174 plays an important role in drug resistance, rapid relapse, and poor outcome of multiple cancers.^34^

We then focused on the function of hsa_circ_0004639 and hsa_circ_0008310, which were supposed to regulate miR‐3174. The results preliminary showed that hsa_circ_0004639 can inhibit malignant biological properties by inhibiting cancer cells’ proliferation, migration, and invasion and hsa_circ_0008310 can promote malignant biological properties.

Our analysis provides new insight into the exploration of pan‐cancer. However, the result is based on bioinformatics and cell function assays. Further exploration and direct mechanism experiments are necessary to confirm the role of these pan‐circRNAs and their networks.

## 5 CONCLUSION

Six circRNAs were identified as pan‐circRNAs, which may involve in carcinogenesis. Among then, the hsa_circ_0004639 can inhibit while the hsa_circ_0008310 can promote malignant biological properties. The ceRNA network was also formed. Enrichment analysis showed it may contribute to the occurrence and development as well as drug resistance of cancers, and the hub genes mainly function in cell division. These analyses may provide new insight into the exploration of pan‐cancer.

## DISCLOSURE

The authors report no conflict of interest.

## AUTHOR CONTRIBUTION

Y. J. and Z. L. conceived and designed the study. Z. C. conducted all experiments and analyzed the data. J. H. and Z. C. contributed to sample collection. Y. J. contributed to funding support. Z. C. wrote the manuscript. Y. J. and Z. L. revised the manuscript.

## Supporting information

Table S1
Table S2
Table S3
Figure S1

## Data Availability

The data is available upon reasonable request.
