# Supplementary material for: Screening and bioinformatics analysis of a ceRNA network based on the circular RNAs, miRNAs, and mRNAs in pan‐cancer
Source: Cancer Med. 2020 Aug 15;9(19):7279–92. doi: 10.1002/cam4.3375 (PMC7541145; doi:10.1002/cam4.3375)

**Table S1.** the basic information of the enrolled patients.

| **Data source** | **ID** | **gender** | **age** | **type** |
| --- | --- | --- | --- | --- |
| GSE101586 | GSM2706423(N)/GSM2706428(T) | female | n.a. | lung adenocarcinoma |
|  | GSM2706424(N)/GSM2706429(T) | female | n.a. | lung adenocarcinoma |
|  | GSM2706425(N)/GSM2706430(T) | female | n.a. | lung adenocarcinoma |
|  | GSM2706426(N)/GSM2706431(T) | female | n.a. | lung adenocarcinoma |
|  | GSM2706427(N)/GSM2706432(T) | female | n.a. | lung adenocarcinoma |
| GSE126095 | GSM3591546(N)/GSM3591556(T) | male | 65 | colorectal cancer |
|  | GSM3591547(N)/GSM3591557(T) | male | 72 | colorectal cancer |
|  | GSM3591548(N)/GSM3591558(T) | female | 67 | colorectal cancer |
|  | GSM3591549(N)/GSM3591559(T) | female | 61 | colorectal cancer |
|  | GSM3591550(N)/GSM3591560(T) | male | 59 | colorectal cancer |
|  | GSM3591551(N)/GSM3591561(T) | male | 74 | colorectal cancer |
|  | GSM3591552(N)/GSM3591562(T) | female | 69 | colorectal cancer |
|  | GSM3591553(N)/GSM3591563(T) | male | 66 | colorectal cancer |
|  | GSM3591554(N)/GSM3591564(T) | male | 56 | colorectal cancer |
|  | GSM3591555(N)/GSM3591565(T) | female | 60 | colorectal cancer |
| GSE79634 | GSM2099896(N)/GSM2099895(T) | male | 67 | pancreatic ductal adenocarcinoma |
|  | GSM2099898(N)/GSM2099897(T) | female | 50 | pancreatic ductal adenocarcinoma |
|  | GSM2099900(N)/GSM2099899(T) | male | 50 | pancreatic ductal adenocarcinoma |
|  | GSM2099902(N)/GSM2099901(T) | male | 62 | pancreatic ductal adenocarcinoma |
|  | GSM2099904(N)/GSM2099903(T) | male | 46 | pancreatic ductal adenocarcinoma |
|  | GSM2099906(N)/GSM2099905(T) | female | 71 | pancreatic ductal adenocarcinoma |
|  | GSM2099908(N)/GSM2099907(T) | male | 49 | pancreatic ductal adenocarcinoma |
|  | GSM2099910(N)/GSM2099909(T) | female | 47 | pancreatic ductal adenocarcinoma |
|  | GSM2099912(N)/GSM2099911(T) | female | 71 | pancreatic ductal adenocarcinoma |
|  | GSM2099914(N)/GSM2099913(T) | female | 72 | pancreatic ductal adenocarcinoma |
|  | GSM2099916(N)/GSM2099915(T) | male | 64 | pancreatic ductal adenocarcinoma |
|  | GSM2099918(N)/GSM2099917(T) | female | 61 | pancreatic ductal adenocarcinoma |
|  | GSM2099920(N)/GSM2099919(T) | male | 60 | pancreatic ductal adenocarcinoma |
|  | GSM2099922(N)/GSM2099921(T) | male | 73 | pancreatic ductal adenocarcinoma |
|  | GSM2099924(N)/GSM2099923(T) | female | 52 | pancreatic ductal adenocarcinoma |
|  | GSM2099926(N)/GSM2099925(T) | male | 61 | pancreatic ductal adenocarcinoma |
|  | GSM2099928(N)/GSM2099927(T) | male | 36 | pancreatic ductal adenocarcinoma |
|  | GSM2099930(N)/GSM2099929(T) | male | 54 | pancreatic ductal adenocarcinoma |
|  | GSM2099932(N)/GSM2099931(T) | female | 60 | pancreatic ductal adenocarcinoma |
|  | GSM2099934(N)/GSM2099933(T) | male | 60 | pancreatic ductal adenocarcinoma |
| GSE83521 | GSM2205874(N)/GSM2205868(T) | n.a. | n.a. | gastric cancer |
|  | GSM2205875(N)/GSM2205869(T) | n.a. | n.a. | gastric cancer |
|  | GSM2205876(N)/GSM2205870(T) | n.a. | n.a. | gastric cancer |
|  | GSM2205877(N)/GSM2205871(T) | n.a. | n.a. | gastric cancer |
|  | GSM2205878(N)/GSM2205872(T) | n.a. | n.a. | gastric cancer |
|  | GSM2205879(N)/GSM2205873(T) | n.a. | n.a. | gastric cancer |
| GSE90737 | GSM2411619 | female | n.a. | cervical tumor |
|  | GSM2411620 | female | n.a. | cervical tumor |
|  | GSM2411621 | female | n.a. | cervical tumor |
|  | GSM2411622 | female | n.a. | cervical tumor |
|  | GSM2411623 | female | n.a. | cervical tumor |
|  | GSM2411624 | female | n.a. | cervical tumor |
|  | GSM2411625 | female | n.a. | cervical tumor |
|  | GSM2411626 | female | n.a. | cervical tumor |
|  | GSM2411627 | female | n.a. | cervical tumor |
|  | GSM2411628 | female | n.a. | cervical tumor |
| GSE93522 | GSM2453884(N)/GSM2453883(T) | female | n.a. | papillary thyroid carcinoma |
|  | GSM2453886(N)/GSM2453885(T) | female | n.a. | papillary thyroid carcinoma |
|  | GSM2453888(N)/GSM2453887(T) | male | n.a. | papillary thyroid carcinoma |
|  | GSM2453890(N)/GSM2453889(T) | male | n.a. | papillary thyroid carcinoma |
|  | GSM2453892(N)/GSM2453891(T) | female | n.a. | papillary thyroid carcinoma |
|  | GSM2453894(N)/GSM2453893(T) | female | n.a. | papillary thyroid carcinoma |
| GSE97332 | GSM2561829(N)/GSM2561836(T) | male | n.a. | hepatocellular carcinoma |
|  | GSM2561830(N)/GSM2561837(T) | male | n.a. | hepatocellular carcinoma |
|  | GSM2561831(N)/GSM2561838(T) | male | n.a. | hepatocellular carcinoma |
|  | GSM2561832(N)/GSM2561839(T) | male | n.a. | hepatocellular carcinoma |
|  | GSM2561833(N)/GSM2561840(T) | male | n.a. | hepatocellular carcinoma |
|  | GSM2561834(N)/GSM2561841(T) | male | n.a. | hepatocellular carcinoma |
|  | GSM2561835(N)/GSM2561842(T) | male | n.a. | hepatocellular carcinoma |
| BC array | 183434 | female | 52 | Breast cancer |
|  | 188293 | female | 42 | Breast cancer |
|  | 188928 | female | 47 | Breast cancer |
|  | S71278 | female | 56 | Breast cancer |
|  | S71504 | female | 35 | Breast cancer |
|  | S73512 | female | 52 | Breast cancer |

**Table S2.** Primers used for qRT-PCR.

| RNA | Primer | Sequennce |
| --- | --- | --- |
| hsa_circ_0004639 | Forward | 5’-GTGTGGAGCAGGTGCCTTAG-3’ |
|  | Reverse | 5’-TGAACTGCAACAGTCTGTGAAC-3’ |
| hsa_circ_0008310 | Forward | 5’-ACTTCATCCACAGGCTCCTC-3’ |
|  | Reverse | 5’-CGGCTCTGCTGCAATACAGG-3’ |
| miR-507 | Forward | 5’-CGCGTTTTGCACCTTTTGG-3’ |
|  | Reverse | 5’-AGTGCAGGGTCCGAGGTATT-3’ |
|  | RT | 5’-GTCGTATCCAGTGCAGGGTCCGAGGTATTCGCACTGGATACGACTTCACT-3’ |
| miR-760 | Forward | 5’-CGCGGCTCTGGGTCTG-3’ |
|  | Reverse | 5’-AGTGCAGGGTCCGAGGTATT-3’ |
|  | RT | 5’-GTCGTATCCAGTGCAGGGTCCGAGGTATTCGCACTGGATACGACTCCCCA-3’ |
| miR-3174 | Forward | 5’-GCGCGTAGTGAGTTAGAGATGC-3’ |
|  | Reverse | 5’-AGTGCAGGGTCCGAGGTATT-3’ |
|  | RT | 5’-GTCGTATCCAGTGCAGGGTCCGAGGTATTCGCACTGGATACGACGGCTCT-3’ |
| miR-4517 | Forward | 5’- CGCGAAATATGATGAAACTCACA -3’ |
|  | Reverse | 5’-AGTGCAGGGTCCGAGGTATT-3’ |
|  | RT | 5’-GTCGTATCCAGTGCAGGGTCCGAGGTATTCGCACTGGATACGACCTCAGC-3’ |

**Table. S3** the protein-coding potential of six pan-circRNAs.

| **circRNA ID** | **ORF site^*^** | | |  | **IRES site^#^** | |
| --- | --- | --- | --- | --- | --- | --- |
|  | **strand** | **start** | **end** |  | **start** | **end** |
| hsa_circ_0008310 | + | 70 | 258 |  | 86 | 259 |
| hsa_circ_0004639 | - | 634 | 215 |  | 51 | 224 |
|  | + | 477 | 755 |  | \ | \ |
|  | + | 124 | 255 |  | \ | \ |
|  | + | 110 | 211 |  | \ | \ |
|  | - | 97 | 2 |  | \ | \ |
|  | + | 505 | 585 |  | \ | \ |
| hsa_circ_0020390 | + | 79 | 168 |  | \ | \ |
|  | + | 187 | 318 |  | \ | \ |
|  | + | 670 | 771 |  | \ | \ |
|  | + | 1573 | 1659 |  | \ | \ |
|  | + | 294 | 1901 |  | \ | \ |
|  | - | 995 | 777 |  | \ | \ |
|  | - | 1744 | 1613 |  | \ | \ |
|  | - | 1213 | 1133 |  | \ | \ |
|  | - | 1000 | 851 |  | \ | \ |
|  | - | 718 | 566 |  | \ | \ |
|  | - | 259 | 149 |  | \ | \ |
|  | - | 78 | 1 |  | \ | \ |
| hsa_circ_0061749 | + | 6 | 140 |  | \ | \ |
| hsa_circ_0076798 | - | 2 | 94 |  | \ | \ |
| hsa_circ_0043256 | + | 5 | 481 |  | \ | \ |
|  | - | 171 | 94 |  | \ | \ |

* The ORF sites were predicted in <https://www.ncbi.nlm.nih.gov/orffinder/>

# The IRES sites were predicted in circbank (<http://www.circbank.cn/>)

**Figure. 1S** the overall survival analysis of the host genes of pan-circRNAs.


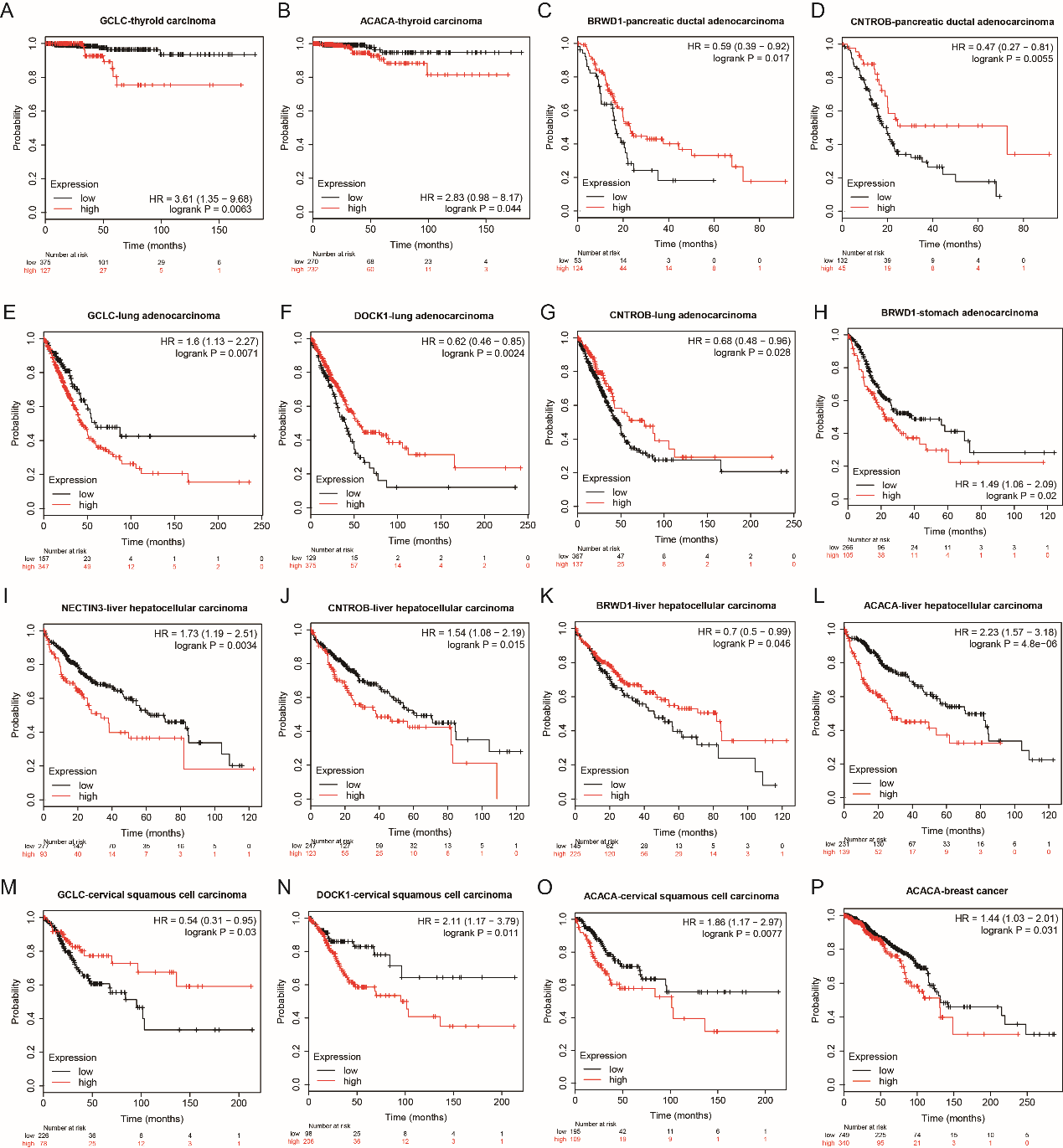

Supplement: Supplementary file 1 — Table S1 Table S2 Table S3 Figure S1 [file CAM4-9-7279-s001.docx]
